# Australian arm of the International Spinal Cord Injury (Aus-InSCI) Community Survey: 3. Drivers of quality of life in people with spinal cord injury

**DOI:** 10.1038/s41393-022-00845-3

**Published:** 2022-08-22

**Authors:** Annette Kifley, Mohit Arora, Andrew Nunn, Ruth Marshall, Timothy Geraghty, Gerard Weber, Sue Urquhart, Ashley Craig, Ian D. Cameron, James W. Middleton

**Affiliations:** 1 John Walsh Centre for Rehabilitation Research, Kolling Institute, Northern Sydney Local Health District, St Leonards, NSW Australia; 2 Sydney Medical School—Northern, Faculty of Medicine and Health, The University of Sydney, Sydney, NSW Australia; 3 Victorian Spinal Cord Service, Austin Health, Heidelberg, VIC Australia; 4 South Australian Spinal Cord Injury Service, Central Adelaide Local Health Network, Adelaide, SA Australia; 5 Faculty of Health and Medical Sciences, University of Adelaide, Adelaide, SA Australia; 6 Queensland Spinal Cord Injuries Service, Division of Rehabilitation, Princess Alexandra Hospital, Brisbane, QLD Australia; 7 The Hopkins Centre, Griffith University, Brisbane, QLD Australia; 8 Spinal Injury Unit, Royal Rehab, Ryde, NSW Australia; 9 Queensland Spinal Cord Injury Service, Princess Alexandra Hospital, Brisbane, QLD Australia; 10 State Spinal Cord Injury Service, Agency for Clinical Innovation, St Leonards, NSW Australia; 11 Spinal Outreach Service, Royal Rehab, Ryde, NSW Australia

**Keywords:** Quality of life, Rehabilitation, Epidemiology, Spinal cord diseases

## Abstract

**Study design:**

Cross-sectional population-based survey for the Australian cohort of the International Spinal Cord Injury (InSCI) Community Survey.

**Objectives:**

To differentiate subgroups of people with spinal cord injury (SCI) who self-report good and poor overall quality of life (QoL) using domains of the International Classification of Functioning (ICF), and to evaluate how these factors contribute to QoL when considered together, while controlling confounders.

**Setting:**

Australian survey data from four state-wide SCI services, one government insurance agency, and three not-for-profit consumer organisations.

**Methods:**

Explanatory factors for QoL were compared between participants reporting poor vs. good QoL. Path models estimated total, direct and mediated contributions from each explanatory factor to QoL ratings after accounting for confounders.

**Results:**

Most participants (62%) reported good or very good QoL, 12% reported poor or very poor QoL. When explanatory factors were considered together, the strongest total effects on QoL involved social integration (+0.36 SDs), subjective social position (+0.29), secondary health condition burden (−0.28), activity/participation problem burden (−0.26), day-to-day assistance (−0.26), mental health (+0.18), pain (−0.16), self-efficacy (+0.15), vitality (+0.14) and environmental barriers (−0.11). Effects of social integration, mental health, vitality, self-efficacy, pain and activity/participation problems were partly or wholly direct.

**Conclusion:**

Opportunities to improve QoL in people with SCI exist at every level of the health system. Virtually all aspects of the ICF framework make a substantive difference to QoL outcomes. Social and psychological factors and ability to complete desired activities have key direct effects and influence effects of secondary health condition burden and environmental barriers.

## Introduction

One of the most important goals of rehabilitation after a spinal cord injury (SCI) is to achieve a satisfactory quality of life (QoL). Published research suggests that basic socio-demographic factors, injury severity and functional limitations are not necessarily the strongest drivers of self-reported overall QoL in people with SCI [1–5]. Studies also suggest that adaptation to injury gradually weakens the relationship between level of functional impairment and perceptions about QoL over time [6, 7].

Multiple international and Australian studies have demonstrated clinically meaningful reductions in QoL among people with SCI in comparison to the general population, with large effect sizes for life satisfaction (~0.75–1) and moderate to large effect sizes for SF-36 subdomains and psychosocial factors (0.3–1 and above) [4, 8–10]. The presence of a large effect size implies that the average difference in QoL between participants with SCI and the general population was large by comparison with a measure of how much QoL varied between individuals, thus implying that the difference was very likely to be important clinically.

The International Spinal Cord Injury (InSCI) survey, and its Australian arm (Aus-InSCI) in particular, allow a detailed examination of factors associated with QoL in a large population-based sample of people with SCI, including domains of the International Classification for Functioning, Health and Disability (ICF) framework (body functions and structure, activities, participation, environmental and personal factors) along with key socio-demographic and injury-related covariables. While facets of the ICF framework are sometimes viewed as aspects of QoL, they can also be viewed as potential drivers and determinants of self-reported overall QoL. In this paper, we take the latter approach in order to investigate how such factors, when considered jointly as a network, may influence self-reported overall QoL.

While previous studies have investigated underlying factors for self-reported QoL from diverse perspectives, and in some cases have examined pathways of effect, relatively few studies considered all aspects of the ICF framework together, in conjunction with adjustment for other key covariables, in a large population-based sample. The aims of this paper are therefore to characterise subgroups of people who self-reported very good or good QoL vs. very poor or poor QoL in terms of components of the ICF framework and to evaluate contributing factors to overall QoL while accounting for potential confounders and mediating factors, using data from the large Aus-InSCI cohort. The analysis will also highlight whether measures addressing specific aspects of the ICF framework may be more likely to translate to subjective QoL improvements among people with SCI, contributing to the identification of priority areas where available resources may best be targeted.

## Methods

Study design and procedures for InSCI and Aus-InSCI have been described previously [11–13]. In brief, Aus-InSCI forms part of the global cross-sectional InSCI study involving 22 countries investigating ‘lived experience’ of people with SCI. Australian residents of at least 18 years of age living in the community with an established traumatic or non-traumatic SCI of at least 12-months duration were involved. Participants were recruited and surveys completed between March 2018 and January 2019. Data from nine data custodians (hospital-based specialised SCI services/units, community organisations and government agencies) across four Australian states were combined for comprehensive representation.

### Measures

The Aus-InSCI questionnaire comprised 193 questions, including socio-demographics, SCI characteristics, body functions and structures, activities and participation, environmental and personal factors, and appraisal of health and wellbeing. Overall QoL within 14 days of interview was self-rated as either very poor, poor, good, very good, or neither poor nor good. Aspects of lived experience were mapped to the five domains of the ICF framework. Level of injury (tetraplegia, paraplegia), completeness of injury (complete, incomplete) and physical health based on a sum score for overall burden of secondary health conditions and maximum pain intensity over past 7 days were mapped to ‘Body Functions and Structure’. A sum score for independence in activities of daily living based on a slightly modified version of the Spinal Cord Independence Measure for Self-Report (m-SCIM-SR), and mobility category (ambulant, self-propelled manual wheelchair use and electric or assisted wheelchair use), were mapped to ‘Activities’. A sum score for overall burden of activity/participation problems was mapped to ‘Activities and Participation’. Current paid work was mapped to ‘Participation’. The Nottwil Environmental Factors Inventory Short Form (NEFI-S) and a sum score for social integration were mapped to ‘Environmental factors’. Psychological factors, including personal psychological factors (in particular, a sum score for perceived injustice and a sum score for self-efficacy) and energy and feelings (SF-36 vitality domain score, SF-36 mental health domain score), were mapped to ‘Personal Factors’. Covariables for the analysis included receiving day-to-day assistance, subjective social position, age at interview, sex, marital status (never married, separated/widowed/divorced, or married/with a partner), living arrangements (living alone, living in an institutional setting, or any other living arrangement), traumatic vs. non-traumatic cause, and time since injury in years. Supplementary Appendix 1 provides further details about survey questions and derived summary measures.

### Statistical methods

Self-reported QoL ratings were categorised into three groups (poor and very poor, good and very good, or neither poor nor good). Characteristics potentially contributing to these QoL categories were examined using means and percentages with 95% confidence intervals (CI). For these analyses, continuous summary measures for explanatory factors were scaled to range from 0 to 100 with the exception of SF-36 vitality and mental health, which are norms-based. These analyses used SAS 9.4 (SAS Institute) and R 3.6.1.

To evaluate aspects of lived experience as potential drivers of self-reported overall QoL, we used path modelling and mediation analyses in M-PLUS version 7.3. The path models consider all explanatory factors together and estimate indirect (mediated), direct (unmediated) and total effects (mediated + unmediated) of each part of the model on overall QoL, after adjusting for potential confounders. Under the ICF framework, the five ICF domains have bidirectional relationships allowing for each aspect of lived experience to influence and be influenced by the other aspects. The path models specify relationships between domains of lived experience and QoL, and they also specify unidirectional relationships between domains of lived experience themselves as a simplifying aspect of the modelling.

**Fig. 1:**
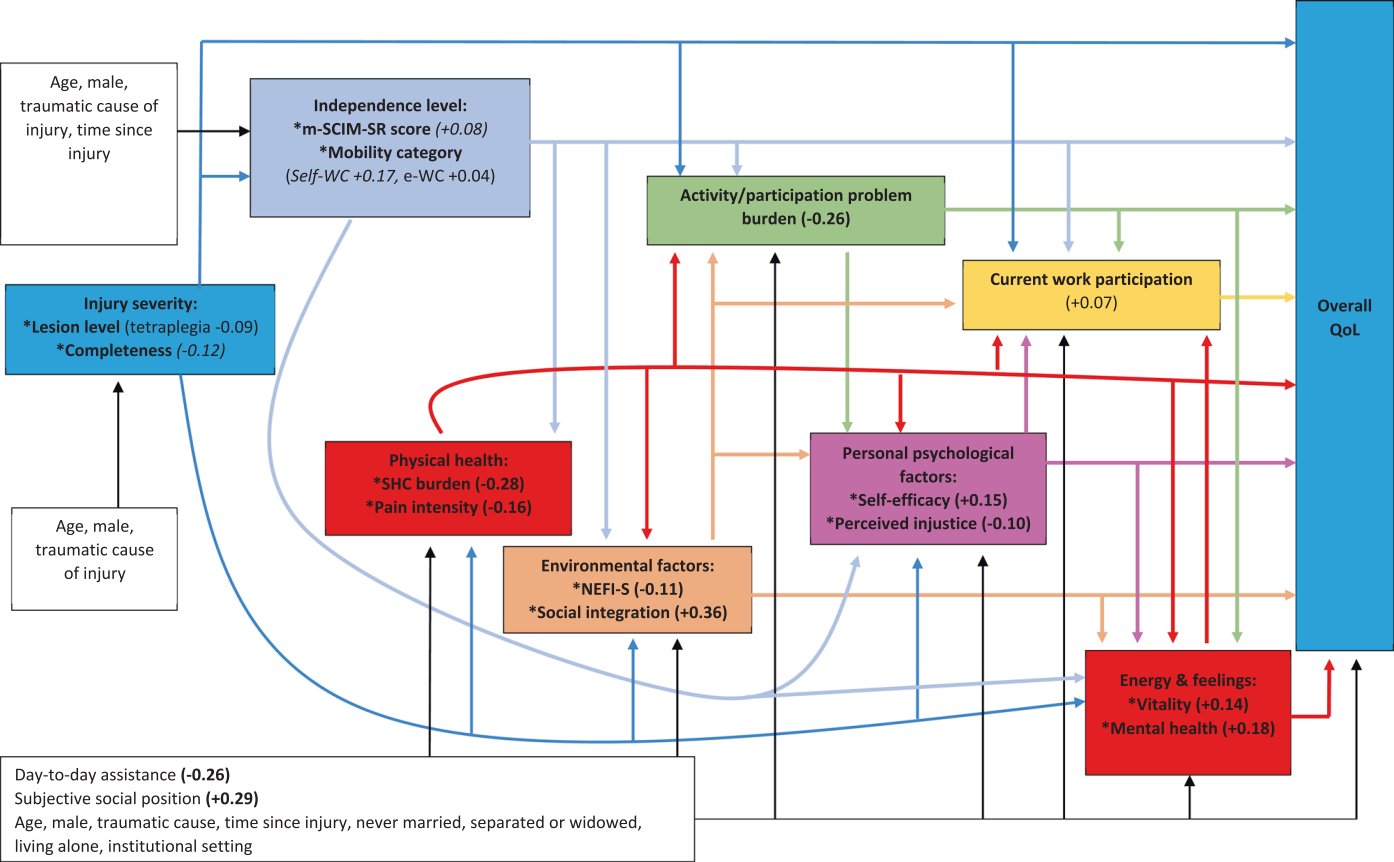
The ICF disability and health framework and self-reported QoL: main path model, and the estimated total effect of each explanatory factor on QoL. Estimated total effects are expressed as the effect of 1 SD of change in a continuous variable, or the presence of a categorical factor, on SDs of change in QoL. Effects in bold indicate *p* < 0.01, italics indicate *p* < 0.05, neither bold nor italic indicates *p* ≥ 0.05. QoL quality of life, m-SCIM-SR modified version of spinal cord independence measure—self-report, SHC secondary health conditions, NEFI-S Nottwil environmental factor index, self-WC use of self-propelled manual wheelchair, e-WC use of electric or assisted wheelchair.

We first hypothesised a main path model based on likely predominant directions of effect (Model 1, Fig. 1). To confirm findings, a series of sensitivity analyses were then used to consider the impact of varying several key aspects of the main model, specifically the role of personal psychological factors, the role of day-to-day assistance, the inclusion of additional relationships between the explanatory factors within ‘Environmental’ and within ‘Energy and feelings’, and the addition of a separate item for ‘Sleep problem severity’ within ‘Physical health’ (Supplementary Appendix 2).

In these analyses, QoL was treated as a continuous variable, and continuous summary measures and QoL were normalised, mean-centred and standardised to standard deviation (SD) 1, except where otherwise stated. Thus, effects identified in the path models are expressed in terms of the effect of a 1 SD change in a continuous explanatory variable on SDs of change in overall QoL. A higher QoL score represented better QoL, therefore a significant positive estimated effect implies an association with better QoL and a significant negative estimated effect implies an association with worse QoL on average. All categorical measures used were dichotomised as indicator variables.

## Results

Aus-InSCI involved 1579 participants with SCI, among whom 1518 answered the overall QoL question. Cohort characteristics have been described previously [5, 11]. In brief, participants were of mean age 57 years (range 19–94 years), predominantly male (73%) with traumatic cause of injury (84%) and living across diverse geographical regions. Their injuries involved incomplete paraplegia (37%), incomplete tetraplegia (30%), complete paraplegia (24%) and complete tetraplegia (9%).

### Characteristics of QoL subgroups

Self-reported overall QoL was poor or very poor in 192 participants (12%), good or very good in 947 participants (62%), and neither good nor poor in 379 participants (25%). There were only modest differences in socio-demographic or injury characteristics between groups, including age, sex and injury characteristics (data not shown). There were only marginal differences in time since injury (mean 15.3, 16.2 and 18.0 years) and age at injury (42.8, 41.1 and 39.5 years) for poor, neither and good QoL groups, respectively. The second paper of this series presents a detailed examination of QoL by socio-demographic factors and injury characteristics in Aus-InSCI participants [5].

Participants with poor vs. good overall QoL reported lower subjective social position (95% CI 3.6–4.3 vs. 5.4–5.7) and were more likely to live alone (95% CI 26–40% vs. 16–22%). They were more likely to be receiving day-to-day assistance (95% CI 82–92% vs. 63–70% for any assistance).

Table 1 shows characteristics of the QoL subgroups based on aspects of function, activities and participation. Participants with poor vs. good overall QoL reported three more severe-extreme secondary health conditions on average, representing ~1 SD of difference, with the most prominent differences being severe-extreme pain (45% difference) and severe-extreme sleep problems (33% difference). Their average m-SCIM-SR scores were ~0.4 SD poorer. They were more likely to use electric or assisted wheelchairs (95% CI 33–48% vs. 19–25%), and less likely to use self-propelled manual wheelchairs (26–34% vs. 34–41%).

**Table 1:** Mean (95% CI) summary measures of functional independence, physical health and activity/participation problem burden, and percentage (95% CI) of participants reporting issues with individual secondary health conditions or activity/participation problems, by QoL subgroup in Aus-InSCI (*n* = 1579).

		Poor QoL (*n* = 192)	Neither (*n* = 379)	Good QoL (*n* = 947)
Summary measures (0–100):	SD	Mean (95% CI)	Mean (95% CI)	Mean (95% CI)
m-SCIM-SR score	28	52.8 (48, 57)	59.6 (56, 63)	64.4 (62, 67)
Secondary health conditions problem burden score	16	46.9 (44.3, 49.6)	38.1 (36.4, 39.7)	28.0 (27.0, 29.0)
Maximum pain intensity	30	68.9 (65.4, 72.5)	57.7 (55.0, 60.5)	42.9 (41.0, 44.8)
Activity/participation problem burden score	22	52.6 (49, 56)	39.1 (37, 42)	21.8 (20, 23)
Individual issues:		% (95% CI)	% (95% CI)	% (95% CI)
Health conditions:				
% reporting severe-extreme problems with:				
Pain		79.3 (72, 85)	59.2 (54, 65)	33.5 (30, 37)
Sexual dysfunction		75.6 (68, 82)	66.2 (61, 71)	53.4 (50, 57)
Sleep problem		54.0 (46,61)	36.1 (31, 42)	21.2 (18, 24)
Contractures		48.7 (41, 56)	38.0 (33, 44)	25.5 (22, 29)
Bladder dysfunction		46.8 (39, 54)	29.6 (25, 35)	19.3 (16, 22)
Muscle spasms or spasticity		46.1 (39, 54)	42.4 (37, 48)	23.7 (21, 27)
Bowel dysfunction		45.8 (38, 53)	32.7 (28, 38)	20.7 (18, 24)
Circulatory problems		39.5 (32, 47)	26.1 (21, 31)	18.0 (15, 21)
Urinary tract infections		32.6 (26, 40)	21.0 (17, 26)	14.9 (12, 18)
Pressure injuries		22.6 (17, 30)	15.2 (11, 20)	8.1 (6, 10)
Autonomic dysreflexia		21.5 (16, 28)	11.7 (8, 16)	7.3 (5, 10)
Postural hypotension		18.8 (13, 26)	13.6 (10, 18)	5.2 (3, 7)
Respiratory problem		16.8 (12, 23)	8.2 (5, 12)	4.4 (3, 6)
Injuries due to loss of sensation		16.3 (11, 23)	11.1 (8, 15)	5.5 (4, 8)
Activity/participation problems:				
% with no problem at all:				
Carrying out daily routine		4.7 (2, 8.7)	11.7 (8.7, 16)	36.6 (33, 40)
Doing things for relaxation and pleasure		11.1 (7, 17)	24.4 (20, 29)	52.9 (49, 57)
Getting household tasks done		11.4 (7, 17)	18.3 (14, 23)	39.3 (36, 43)
Handling stress		12.2 (8, 18)	13.1 (10, 17)	39.8 (36, 43)
Getting where you want to go		15.3 (10, 21.2)	25. 3 (21.2, 30)	52.3 (49, 56)
Intimate relationships		18.4 (13, 25)	24.9 (20, 30)	44.4 (41, 48)
Providing care and support to others		19.7 (14, 27)	29.0 (24, 34)	46.8 (43, 51)
Looking after health, food, exercise, medicines		23.8 (18, 31)	38.5 (33, 44)	62.7 (59, 66)
Using public transport		27.5 (21, 35)	43.4 (38, 49)	58.4 (55, 62)
Interacting with people		36.2 (29, 44)	38.8 (34, 44)	71.2 (68, 74)
Using private transportation		36.3 (29, 44)	51.5 (46, 57)	71.5 (68, 75)
% with severe-extreme problem:				
Carrying out daily routine		51.3 (40, 65)	23.6 (17, 31)	8.5 (6, 12)
Doing things for relaxation and pleasure		48.7 (37, 62)	22.3 (16, 30)	6.2 (4, 9)
Getting household tasks done		53.5 (41, 68)	33.2 (26, 42)	11.4 (8, 15)
Handling stress		38.1 (28, 51)	23.9 (18, 32)	7.2 (5, 10)
Getting where you want to go		48.7 (37, 62)	25.1 (19, 33)	10.2 (7, 14)
Intimate relationships		66.5 (54, 80)	46.3 (38, 56)	24.1 (20, 29)
Providing care and support to others		50.6 (38, 65)	36.5 (29, 46)	16.2 (12, 21)
Looking after health, food, exercise, medicines		27.0 (18, 39)	11.5 (7, 18)	4.2 (2, 7)
Using public transport		50.3 (40, 63)	36.5 (29, 46)	21.2 (17, 26)
Interacting with people		25.5 (17, 37)	16.2 (11, 23)	3.0 (1, 5)
Using private transportation		24.7 (16, 36)	14.7 (10, 21)	7.4 (5, 11)

*m-SCIM-SR* modified Spinal Cord Independence Measure—Self-Report, *SHC* secondary health conditions, *SD* standard deviation, *CI* confidence interval.

Participants with poor QoL reported at least one severe-extreme activity/participation problem in 94% of cases, compared to 50% of participants with good QoL. Their overall activity/participation problem burden score was almost 1.5 SD higher. The most prominent differences in severe-extreme activity/participation problems involved doing things for relaxation or pleasure (43% difference), getting household tasks done (43% difference), intimate relationships (43% difference), and carrying out daily routine (42% difference). Current participation in paid work was 20% lower (95% CI 10–21% vs. 31–38%).

Table 2 shows characteristics of the QoL subgroups by social, psychological and environmental factors. Participants with poor vs. good QoL felt less positive on personal psychological factors by ~1.25 SD, and reported lower vitality and mental health scores by ~1.25–1.5 SDs on average, with perceived injustice ~1 SD higher and social integration ~1.25 SDs lower. They reported greater environmental barriers to participation, with NEFI-S scores 20 points higher on average, representing ~1 SD of difference. The most prominent differences in individual barriers reported to make life a lot harder were access to homes (28% difference), financial problems (27% difference) and unfavourable climactic conditions (26% difference). Few participants rated their overall QoL as ‘good or very good’ if they felt that attitudes of others or lack of services, supplies, devices or technology were a major issue.

**Table 2:** Mean (95% CI) summary measures of social, psychological and environmental factors, and percentage (95% CI) of participants reporting issues with individual social, psychological and environmental factors, by QoL subgroup.

		Poor QoL (*n* = 192)	Neither (*n* = 379)	Good QoL (*n* = 947)
Summary measures (0–100):	SD	Mean (95% CI)	Mean (95% CI)	Mean (95% CI)
Nottwil Environmental Factor Inventory score	22	48.9 (46,0, 51.8)	39.8 (37.5, 42.0)	28.0 (26,7, 29.4)
Personal psychological factors sum score	19	43.6 (41, 47)	50.3 (48, 52)	68.9 (67, 70)
Social integration sum score (excludes work item)	16	54.5 (52, 57)	61.7 (60, 64)	75.1 (74, 76)
Perceived injustice sum score	26	67.5 (64, 71)	50.3 (48, 52)	40.1 (38, 42)
Summary measures (norms-based):				
SF36 Vitality domain score	12	31.5 (30.1, 32.9)	37.4 (36.4, 38.3)	46.4 (45.7, 47.1)
SF36 Mental health domain score	12	32.3 (30.5, 34.1)	39.7 (38.6, 40.9)	50.5 (49.9, 51.2)
Individual issues:		% (95% CI)	% (95% CI)	% (95% CI)
Environmental barriers:				
% reporting ‘made my life a lot harder’:				
Access to homes		44.4 (37, 52)	27.3 (23, 32)	16.4 (14, 19)
Climactic conditions		37.9 (31, 45)	20.1 (16, 25)	12.1 (10, 15)
Financial problems		35.1 (28, 43)	23.5 (19, 29)	8.4 (6, 11)
Access to public places		29.3 (23, 37)	16.3 (12, 21)	10.9 (9, 14)
Insufficient state services		27.0 (21, 34)	16.1 (12, 21)	5.9 (4, 8)
Lack of means to travel long distances		25.8 (20, 33)	18.9 (15, 24)	10.0 (8, 13)
Lack of devices or technology to travel short distances		16.9 (12, 23)	6.9 (4.7, 10)	3.5 (2, 5)
Societal attitudes		16.8 (12, 23)	10.0 (7, 14)	2.9 (2, 5)
Attitudes of family and/or relatives		13.1 (9, 19)	5.6 (3.6, 9)	1.3 (0.7, 3)
Attitudes of friends		13.1 (9, 19)	6.4 (4, 10)	1.1 (0.5, 2)
Insufficient communication devices		11.1 (7, 17)	5.7 (3, 9)	1.3 (0.7, 3)
Lack of nursing care or support services		10.9 (7, 17)	9.1 (6, 13)	2.7 (1, 4)
Attitudes of others, e.g. neighbours, colleagues		10.6 (6, 16)	6.2 (4, 10)	1.2 (0.6, 3)
Lack of medication, medical aids, supplies		6.3 (3, 11)	5.4 (3, 9)	1.7 (1, 3)
Social integration:				
% in agreement with statement:				
Cooperative co-workers		17.5 (10, 25)	25.1 (20, 31)	42.4 (38, 47)
Close to other local people		28.2 (21, 35)	31.7 (26, 37)	55.2 (52, 59)
Control over own life		39.2 (32, 47)	61.4 (56, 67)	89.2 (87, 92)
Little chance to show how capable you are		48.1 (40, 56)	27.3 (22, 32)	16.7 (14, 20)
Treated with respect		64.6 (57, 72)	66.5 (61, 72)	88.9 (86, 91)
Receive help and support from people close to you		75.5 (69, 82)	80.7 (76, 85)	90.4 (88, 93)
Personal psychological factors: self-efficacy, optimism, autonomy, belonging:				
% in agreement with statement:				
Able to achieve dreams, hopes and wishes		8.4 (4, 13)	14.1 (10, 18)	42.4 (39, 46)
Confident of maintaining good health		22.2 (16, 29)	30.6 (25, 36)	62.0 (58, 66)
Worried about the future		77.4 (71, 84)	75.5 (71, 80)	50.0 (46, 54)
Injury makes you stronger		27.2 (20, 34)	32.0 (27, 37)	62.7 (59, 66)
Confident of dealing with unexpected events		31.2 (24, 38)	33.1 (28, 38)	68.3 (65, 72)
Confident of overcoming opposition		36.3 (29, 44)	41.2 (36, 47)	68.4 (65, 72)
Feel included when among other people		38.7 (31, 46)	43.7 (38, 49)	76.7 (73, 80)
Confident of maintaining contact with important people		50.3 (43, 58)	59.1 (54, 65)	86.1 (83, 89)
Control over major decisions		51.3 (44, 59)	64.4 (59, 70)	83.2 (80, 86)
Personal psychological factors: perceived injustice:				
% reporting applies most or all of the time:				
I just want my life back		81.3 (75, 87)	68.8 (64, 74)	38.3 (35, 42)
Most people don’t understand severity of condition		76.5 (70, 83)	66.9 (62, 72)	54.8 (51, 58)
It all seems so unfair		65.2 (58, 73)	47.4 (42, 53)	20.4 (17, 24)
Suffering due to someone else’s negligence		36.2 (29, 44)	26.7 (22, 32)	15.1 (12, 18)

*SD* standard deviation, *CI* confidence interval.

### Drivers of overall QoL

Table 3 and Fig. 1 display findings of the main path model (Model 1). When explanatory factors were considered together in the main model, the strongest total effects on QoL involved social integration (total effect +0.36 SDs, meaning that higher social integration was associated with better QoL), subjective social position (+0.29), secondary health conditions problem burden (−0.28, meaning that higher secondary health conditions problem burden was associated with poorer QoL), activity/participation problem burden (−0.26), day-to-day assistance (−0.26), mental health (+0.18), pain severity (−0.16), self-efficacy (+0.15), vitality (+0.14) and NEFI-S environmental barriers to participation (−0.11). Of these, social integration, mental health, vitality, self-efficacy, pain and activity/participation problem burden included direct (unmediated) effects, while effects of subjective social position, secondary health conditions problem burden, day-to-day assistance and NEFI-S were predominantly indirect (mediated). Effects of day-to-day assistance and subjective social position remained the same after adjustment for other covariates in the main model (data not shown).

**Table 3:** Total, direct and indirect effects of ICF disability and health framework measures on overall QoL ratings under the main path model.

Effects on QoL (expressed as SDs of change)	Direct effect estimate (95% CI)	*p* value	Total effect estimate (95% CI)	*p* value	Indirect effect estimate (95% CI)	*p* value
Current work participation:						
Current paid work	0.07 (−0.02, 0.16)	0.12	0.07 (−0.02, 0.16)	0.12	n/a	n/a
Energy and feelings:						
SF-36 Mental health domain score (per SD)	0.18 (0.12, 0.24)	**<0.001**	0.18 (0.12, 0.24)	**<0.001**	−0.001 (−0.004, 0.002)	0.4
SF-36 vitality domain score (per SD)	0.14 (0.08, 0.2)	**<0.001**	0.14 (0.08, 0.2)	**<0.001**	−0.001 (−0.004, 0.001)	0.4
Personal psychological factors:						
Self-efficacy sum score (per SD)	0.09 (0.04, 0.15)	**<0.001**	0.15 (0.09, 0.2)	**<0.001**	0.05 (0.03, 0.07)	**<0.001**
Perceived injustice sum score (per SD)	−0.05 (−0.1, −0.003)	**0.04**	−0.10 (−0.15, −0.05)	**<0.001**	−0.05 (−0.07, −0.03)	**<0.001**
Activities and participation:						
Activity/participation problem burden score (per SD)	−0.12 (−0.18, −0.05)	**<0.001**	−0.26 (−0.32, −0.09)	**<0.001**	−0.14 (−0.17, −0.11)	**<0.001**
Environmental factors:						
NEFI-S (per SD)	−0.04 (−0.09, 0.02)	0.14	−0.11 (−0.17, −0.06)	**<0.001**	−0.08 (−0.11, −0.04)	**<0.001**
Social integration sum score (per SD)	0.19 (0.13, 0.24)	**<0.001**	0.36 (0.31, 0.41)	**<0.001**	0.18 (0.14, 0.21)	**<0.001**
Physical health:						
Secondary health conditions burden score (per SD)	0.007 (−0.05, 0.07)	0.8	−0.28 (−0.34, −0.22)	**<0.001**	−0.28 (−0.33, −0.24)	**<0.001**
Maximum pain intensity (per SD)	−0.06 (−0.1, −0.01)	**0.006**	−0.16 (−0.21, −0.11)	**<0.001**	−0.10 (−0.13, −0.07)	**<0.001**
Independence level:						
m-SCIM-SR (per SD)	−0.02 (−0.09, 0.05)	0.5	0.08 (0.01, 0.16)	**0.03**	0.10 (0.05, 0.15)	**<0.001**
Use of electric or assisted wheelchair	−0.05 (−0.19, 0.08)	0.4	0.04 (−0.13, 0.2)	0.6	0.09 (−0.02, 0.2)	**0.09**
Use of self-propelled manual wheelchair	0.04 (−0.08, 0.16)	0.5	0.17 (0.03, 0.31)	**0.02**	0.13 (0.03, 0.22)	**0.004**
Injury severity:						
Complete lesion	−0.17 (−0.26, −0.07)	**<0.001**	−0.12 (−0.23, −0.01)	**0.02**	0.05 (−0.04, 0.14)	0.2
Tetraplegia	−0.04 (−0.13, 0.05)	0.4	−0.09 (−0.19, 0.005)	0.06	−0.05 (−0.13, 0.02)	0.16
Assistance:						
Any day-to-day assistance	−0.02 (−0.12, 0.08)	0.6	−0.26 (−0.38, −0.14)	**<0.001**	−0.24 (−0.32, −0.16)	**<0.001**
Socioeconomic status:						
Subjective social position ladder (per SD)	0.03 (−0.01, 0.08)	0.14	0.29 (0.24, 0.34)	**<0.001**	0.26 (0.22, 0.3)	**<0.001**
Covariables:						
Traumatic SCI	0.05 (−0.06, 0.16)	0.3	0.07 (−0.06, 0.19)	0.3	0.02 (−0.07, 0.1)	0.7
Time since injury in years (10 years)	0.03 (0.01, 0.06)	**0.02**	0.08 (0.04, 0.11)	**<0.001**	0.04 (0.02, 0.07)	**<0.001**
Age in years (10 years)	−0.06 (−0.09, −0.03)	**<0.001**	−0.03 (−0.06, 0.01)	0.1	0.03 (0.01, 0.06)	**0.01**
Male	−0.12 (−0.2, −0.03)	**0.006**	−0.03 (−0.14, 0.06)	0.5	0.08 (0.01, 0.15)	**0.01**
Never married	−0.13 (−0.24, −0.01)	**0.02**	−0.14 (−0.28, −0.005)	**0.04**	−0.01 (−0.1, 0.08)	0.8
Separated, divorced or widowed	−0.04 (−0.16, 0.09)	0.5	−0.12 (−0.27, 0.03)	0.1	−0.08 (−0.18, 0.06)	0.07
Living alone at home	−0.11 (−0.22, 0.01)	0.051	−0.06 (−0.2, 0.08)	0.4	0.05 (−0.04, 0.13)	0.2
Living in an institutional setting	0.11 (−0.11, 0.32)	0.3	0.21 (−0.06, 0.48)	0.13	0.1 (−0.06, 0.27)	0.2

Continuous variables included quality of life (QoL), variables under energy and feeling, personal psychological factors, activities and participation, environmental factors and physical health, the modified Spinal Cord Independence measure—Self-Report (m-SCIM-SR), subjective social position ladder, time since injury and age. Dichotomous variables included current paid work, use of an electric or assisted wheelchair, use of a self-propelled manual wheelchair, having a complete lesion, tetraplegia, receiving any day-to-day assistance, male sex, being never married, being separated/divorced or widowed, living alone at home, and living in an institutional setting.

Bold text indicates *p* < 0.05.

*SD* standard deviation, *CI* confidence interval.

Supplementary Appendix 2 displays details of the sensitivity analyses. In brief, if self-efficacy and perceived injustice were treated as underlying influences on secondary health conditions problem burden, pain severity, activity/participation problem burden, NEFI-S and social integration, then the total effects of self-efficacy and perceived injustice on QoL were strengthened, while total effects of secondary health conditions problem burden, pain, activity/participation problem burden, social integration and NEFI-S were reduced but not eliminated.

If possible influences of injury characteristics, functional independence and physical health on day-to-day assistance were included in the model, then the total effect of m-SCIM-SR score on QoL nearly doubled. Day-to-day assistance was more strongly associated with independence level than with physical health. Together, these findings suggest that independence level influences QoL ratings substantively, and that a large part of the impact of day-to-day assistance in the main model reflects this.

Indirect effects of NEFI-S and vitality on QoL strengthened if paths were added for an impact of NEFI-S on social integration and vitality on mental health. Sleep problem severity had a substantive negative total effect, predominantly indirect, if added alongside secondary health conditions and pain under ‘Physical health’.

## Discussion

In this paper, we have proposed and examined a model for drivers of QoL that is grounded both in the ICF framework and in participant’s own perception of QoL. Virtually all aspects of the ICF framework made a material difference to the self-reported QoL of people with SCI. Social and psychological factors had key direct impacts and were also key mediators of burdens related to secondary health conditions, difficulty in completing desired activities and environmental barriers. Activity/participation problem burden and pain intensity also directly affected QoL, even after considering all other factors. While functional independence level was important, other aspects of the ICF framework had a greater overall impact on QoL. Primary analyses and sensitivity analyses both support these messages.

These findings underline opportunities to improve QoL outcomes for people with SCI at every level of the health system and suggest that improvements in each and any of the ICF domains are likely to be worthwhile in improving QoL. At system level, participants clearly struggled if access to services, care, assistive devices or supplies was a barrier, with very few participants reporting good QoL in these circumstances. At community level, the importance of community acceptance and inclusiveness is highlighted, with very few participants reporting good QoL if they perceived that the attitudes of people around them was a barrier. The negative impact of social attitudes and issues with access to care and services is supported by the 2020 international report from the InSCI survey across 22 countries, in which the health system indicator showing the strongest association with overall QoL was social attitudes, followed by access to health care, nursing care and access to public spaces. These issues were not limited to any single participating country, although country-specific issues for improvement varied [14]. At an individual level, the path analyses emphasise the role of social and psychological factors.

Substantial existing literature supports our finding of central roles for social and psychological factors as drivers of QoL ratings in people with SCI [9]. In relation to social factors, Whalley Hammell, for example, concluded in a qualitative review (2004) that dissatisfaction with life after SCI arises substantially due to social disadvantage [3]. Post and van Leeuwen in their review identified that emotion-focussed social support was more strongly related to life satisfaction than problem-focussed social support, that quality rather than quantity of social support was more strongly associated with mental health, and that mental health was most strongly associated with social integration and reassurance of worth compared with other subscales of the Social Provisions Scale [9]. Other recent studies have examined links between life satisfaction and loneliness, relationship quality, social network intimacy, and peer support [15–18]. A path analysis in an African sample of people with SCI found that motor function, sensory function, self-esteem, functional independence level and social support all contributed to QoL, with the largest contribution from social support [19]. Their finding mirrors ours on the importance of social integration. From the psychological perspective, reviews of empirical evidence on the connections between personal psychological factors and QoL indicate that self-efficacy, perceived control, resilience, motivation/purpose in life, and hope are consistently associated with life satisfaction and wellbeing, and social skills, self-efficacy and personality traits are associated with better mental health and higher participation [2, 20].

Thus, both our findings and other accumulating evidence indicate that social and psychological factors substantially influence QoL and life satisfaction in people with SCI. Where the quality of social connections and personal relationships can be strengthened, the strongest individual effect on QoL seen in our main path model was that of social integration. Where psychological resources, personal outlook and aspects of mental health can be strengthened, these improvements would translate not only to direct benefits but also indirect ones since these factors mediated perceived burdens from other issues and the response to them.

Our findings also support an influence of perceived injustice, alongside self-efficacy, on QoL in people with SCI. Perceived injustice is associated with adverse QoL and mental health outcomes in trauma care settings and in people with pain [21, 22]. It may represent a core appraisal influencing adjustment after SCI, and a potentially modifiable target for intervention to improve QoL [23].

Our finding that effects of overall burden of secondary health conditions on QoL were substantially indirect after accounting for injury characteristics, functional independence level, mobility and general covariables accords with a concentric biopsychosocial model for QoL. The concentric biopsychosocial model specifies that social factors mediate or moderate the effects of biological factors on QoL, and psychological factors mediate or moderate the effects of both biological and social factors. Notably, individual conditions such as pain demonstrated direct effects in addition to indirect effects when modelled separately from the global burden score. Smedema evaluated a concentric biopsychosocial model centred around life satisfaction in 235 people with SCI in the United States and also found that psychosocial factors mediated effects of pain on life satisfaction substantially, but not entirely [24]. Muller et al. found a mediating role of participation in the effect of pain on depressive mood and QoL, which did not apply across the board but depended on time since injury, severity and completeness [25].

Several recent Canadian studies have examined the role of secondary health conditions alongside other factors in people with SCI. Rivers et al. used path modelling to examine the effects of secondary health conditions on function, health-related QoL and life satisfaction. At each stage of their path model, the number of secondary health conditions had negative effects, not only on function, but also on QoL and life satisfaction [7]. Sweet et al. used structural equation modelling to examine jointly the effect on QoL of latent constructs representing unmet vital needs (health care, financial care, transport, housing and equipment), unmet developmental needs (peer support, job training, recreation, counselling), neurophysiological symptoms (sleep, spasticity, autonomic dysreflexia, fatigue, dizziness), bowel and bladder dysfunction, sexual dysfunction and pressure ulcers. Significant effects were found for both types of unmet need and for neurophysiological symptoms [26]. Engel et al. reported negative total effects of SHC on subjective wellbeing, largely or entirely mediated via capability wellbeing (measured via five domains for feeling settled and secure, having love friendship and support, being independent, achievement/progress, and enjoyment/pleasure) and health-related QoL (measured via three physical domains including pain and five psychosocial domains for mental health, happiness, self-worth, coping and relationships) [27].

While these path models cannot be directly compared with ours, a consistent message is that secondary health conditions are important alongside other factors and that mediating factors are involved in this effect. Our finding that overall burden of secondary health conditions and NEFI-S scores showed mainly indirect effects on overall QoL suggests that their impacts can be mitigated if social, psychological and mental health factors and ability to participate in desired activities can be maintained.

Our study has several limitations. Inter-relationships between factors under the ICF framework are complex and bidirectional. It is not possible to make causal inferences about these relationships from cross-sectional data. Therefore, in this paper, the interpretation of different aspects of lived experience as potential determinants of QoL, and as potential mediating factors, is a conceptual interpretation based on a theoretical framework for QoL, rather than being an evidence based conclusion drawn from the analysis of the cross-sectional data. While our suggested path models are not definitive, nonetheless the evaluation of a reasonable hypothesised model in conjunction with key sensitivity analyses is informative. The hypothesised path model draws on key aspects of the ICF framework, biopsychosocial models for wellbeing and health-related QoL and the Spinal Cord Injury Adjustment Model to reflect a conceptual framework for potential determinants of QoL [28, 29]. In future work, we will be able to examine the temporal nature of relationships under the model using data from the longitudinal phase of Aus-InSCI. This will allow further confirmation of effect direction.

Our path model places current work participation at the most proximate location to QoL, and this aspect of the modelling is likely oversimplified, in so far as work participation potentially fosters positive settings in the rest of the framework, such as positive subjective social position and financial resources, social connections, positive outlook, sense of purpose and sense of self-worth and self-efficacy. Indeed, in a systematic review of qualitative studies, Hilton et al. concluded that there is an intrinsic need for occupational engagement which generally outweighs difficulties involved in regaining work after SCI, and that timely reassurance, support and assistance about the possibilities and value of work provide injured people with a positive message about their future [30]. Therefore, although our analysis did not show a further positive effect of current work participation on overall QoL ratings when all other aspects of the ICF framework were already accounted for, we would not infer any contradiction of other literature supporting positive benefits from work participation.

Our results arise in a relatively high resource setting, where investments in disability care and support have been in focus politically and there is an ongoing open public discourse about supporting the participation and integration of people with disability in society. It is unknown how well the findings would generalise to other contexts where the relative weight of issues may differ. However, the key message that biological, environmental, social and psychological dimensions of lived experience were all impactful on perceived QoL of people with SCI when taken together is unlikely to be particular to only one resource setting or societal context. Patterns of contributing factors to QoL may be different for different subgroups of the population with SCI, which will require future investigation. Data from other countries participating in InSCI will enable us to explore these relationships further.

For our QoL outcome measure in our path models we used the single question of how participants rated their overall QoL on five response levels, as a continuous measure. The key advantage and strength of this approach is that each person’s response is based on their own perception of what overall QoL means [3, 28]*.* A potential disadvantage is that this measure would have relatively low precision, however, the large cohort size supports this choice for analysis.

## Conclusion

Opportunities to improve QoL outcomes for people with SCI exist at every level of the health system, based on a holistic approach that includes attention to social and psychological factors and mental health. We demonstrate substantial links between each ICF domain and self-reported QoL in people with SCI using a model for drivers of QoL based on the ICF framework and considering all ICF domains simultaneously. Longitudinal data from the next phase of Aus-InSCI will enable us to examine these relationships more deeply, refining this approach and clarifying the temporal nature of relationships between ICF domains and reported QoL.

## Supplementary information

Supplementary Material

## Data Availability

De-identified data are available upon request and with permission gained from the Aus-InSCI Community Survey National Scientific Committee.
