# Supplementary material for: Australian arm of the International Spinal Cord Injury (Aus-InSCI) Community Survey: 3. Drivers of quality of life in people with spinal cord injury
Source: Spinal Cord. 2022 Aug 22;61(3):185–93. doi: 10.1038/s41393-022-00845-3 (PMC10023557; doi:10.1038/s41393-022-00845-3)
Supplement: Supplementary file 1 — Supplementary Material [file 41393_2022_845_MOESM1_ESM.pdf]

SUPPLEMENTARY MATERIAL

Supplementary Appendix 1: Survey questions and derived summary measures

Quality of life (QoL) measures

Self-rated overall QoL within 14 days of interview was rated on a five-point scale, as either very poor, poor, neither poor nor good, good or very good. This question was taken from the WHOQOL-BREF QoL assessment tool [1].

The interpretation of the middle option 'Neither good nor poor' is open to interpretation, and selection of this option may mean genuine neutrality, ambivalence (indifference or conflict), lack of cognitive effort or willingness to give a meaningful answer, or avoidance of responses that may be seen as embarrassing or undesirable. It has been recently suggested that it is often better to allow for such a middle option in survey questions and, in the context of this paper, any undesirable impact on data quality is likely to be very small [2].

ICF framework and related measures

We mapped aspects of lived experience captured in the Aus-InSCI survey to the five core domains of the ICF framework in following way. Level and completeness of injury, and physical health based on secondary health problems and pain were mapped to 'Body Function'. Independence in activities of daily living and mobility category were mapped to 'Activities', while activity/participation problem burden related to instrumental activities of daily living, doing things for relaxation and pleasure, interacting with people and intimate relationships was mapped to 'Activities and Participation'. Paid work was mapped to 'Participation' and was handled separately from activity/participation problem burden. Environmental barriers and social integration were mapped to 'Environmental factors'. Psychological factors, including personal psychological factors (perceived injustice and self-efficacy) and energy and feelings (vitality, mental health), were mapped to 'Personal factors'.

Under the ICF framework, the 'Personal factors' domain is not rigidly defined and allows for inclusion of a range of characteristics and issues that may not be fully captured elsewhere. 'Personal factors' may include sociodemographic personal characteristics, internal reactions (such as thoughts, feelings, beliefs and motivations), and patterns of behaviour and experience (such as coping styles, personality traits and lifestyle choices), while 'Body Functions' allows for inclusion of physical, mental and psychological functions [3]. In this paper, we have considered psychological factors together under one umbrella, including personal psychological factors and mental health, and have mapped all of these to 'Personal factors'. We acknowledge that mental and physical function are closely interconnected, and both could alternatively have been mapped to 'Body Functions'.

Participants self-reported their injury level (dichotomised as tetraplegia versus paraplegia) and completeness (dichotomised as not able (complete) versus able (incomplete) to feel or move any part of the body below injury level).

A sum score for secondary health conditions (SHC) problem burden took into account the number and severity of self-reported secondary health problems reported over the last 3 months for 14 specific issues, including sleep problems, bowel dysfunction, urinary tract infections, bladder dysfunction, sexual dysfunction, contractures, muscle spasms/spasticity, pain, pressure sores or ulcers, respiratory problems, injury caused by loss of sensation, circulatory problems, autonomic dysreflexia and postural hypotension. These were rated on a five-point scale from 'No problem' to 'Extreme problem'. These questions represent small modifications of the Spinal Cord Injury Secondary Conditions Scale in that participants

were asked to rate the severity of health problems for the 14 selected issues [4]. Maximum pain intensity over the last week was rated on an 11-point scale from 'No pain' to 'Pain as bad as you can imagine'.

An overall score for independence in activities of daily living (m-SCIM-SR) was ascertained from an abbreviated and slightly modified version of Spinal Cord Independence Measure for self-report [5]. This included 12 questions covering self-care, sphincter management, use of the toilet, and three mobility questions (ability to perform four activities unassisted, and degree of independence in transferring from bed to a wheelchair, and in moving moderate distances of 10-100 metres).

Three mobility categories were defined based on degree of independence in moving moderate distances: ambulant with or without assistance while walking, use of a self-propelled manual wheelchair and use of an electric wheelchair or requiring partial assistance to operate a manual wheelchair or requiring total assistance.

Severity of activity/participation problems over the last 4 weeks was rated on a five-point scale from no problem to extreme problem for 11 activities including carrying out daily routine, handling stress, getting where you want to go, using public and private transportation, looking after your health, getting household tasks done, providing care or support to others, interacting with people, intimate relationships and doing things for relaxation or pleasure. A sum score for activity/participation problem burden was defined taking into account the number and severity of these problems, allowing up to two items to be missed by the participant.

Participants self-reported current engagement in paid work (PW).

The Nottwil Environmental Factors Inventory Short Form (NEFI-S) evaluated environmental barriers to participation in society over the past four weeks [6]. A list of fourteen barriers covering issues such as accessibility to public places and homes, climatic conditions, social attitudes, financial difficulties, and the lack of transportation, devices, nursing care, support services, medications, medical aids and supplies, communication devices or state services were rated either as 'Not applicable', 'No influence', 'Made life a little harder' or 'Made life a lot harder'.

A sum score for social integration was based on five questions. The first two assessed level of agreement with 'having little chance to show how capable I am' and 'feeling close to people in my area' on a five-point scale from 'Agree strongly' to 'Disagree strongly'. The remaining three assessed level of agreement with 'feeling treated with respect', 'receiving help and support from people close to you' and 'having control over own life' on a seven-point scale from 'Not at all' to 'A great deal' and from 'No control' to 'Extreme control'. These questions derive from the European Social Survey.

The SF-36 vitality and mental health domains were used to measure energy and feelings over the last four weeks. Norms-based domain scores used Australian reference values [7].

A sum score for self-efficacy was based on level of agreement with four statements about how participant saw themselves, rated on a five-point scale from 'Not at all' to 'Completely'. These addressed confidence in dealing with opposition and unexpected events, maintaining important contacts and good health, and making the big decisions in life. These questions derive from the Moorong Self-Efficacy Scale (MSES) and the General Self-Efficacy Scale (GSES) [8, 9].

A sum score for perceived injustice was based on level of agreement with four statements, rated on a five-point scale from 'Not at all' to 'Completely'. These statements were that 'most people do not understand how severe my condition is', 'I am suffering because of someone else's negligence', 'I just want my life back', and 'it all seems so unfair'. These questions are selected from the Injustice Experience Questionnaire [10].

Covariables

The following ten measures were considered as covariables in the analysis. Receiving day-to-day assistance from family, friends, professionals or paid assistance was dichotomised as yes or no for any assistance. Socioeconomic status was ascertained as subjective social position on a 10-point ladder indicating how participants rated their overall social standing based on having money, education and respected work. Other covariables included age at interview, sex, marital status (single, separated/divorced/widowed versus married or with a partner), household composition (living alone, living in an institution, living with other adults and/or children), traumatic versus non-traumatic cause of injury, time since injury.

References

1. WHOQOL-BREF: Introduction, administration, scoring and generic version of the assessment. World Health Organization. Geneva; 1996.
2. Wang R, Krosnick JA. Middle alternatives and measurement validity: A recommendation for survey researchers. International Journal of Social Research Methodology: Theory & Practice. 2020;23(2):169-84.
3. Geyh S, Müller R, Peter C, Bickenbach JE, Post MW, Stucki G, et al. Capturing the psychologic-personal perspective in spinal cord injury. Am J Phys Med Rehabil. 2011;90(11 Suppl 2):S79-96.
4. Kalpakjian CZ, Scelza WM, Forchheimer MB, Toussaint LL. Preliminary reliability and validity of a Spinal Cord Injury Secondary Conditions Scale. J Spinal Cord Med. 2007;30(2):131-9.
5. Fekete C, Eriks-Hoogland I, Baumberger M, Catz A, Itzkovich M, Lüthi H, et al. Development and validation of a self-report version of the Spinal Cord Independence Measure (SCIM III). Spinal Cord. 2013;51(1):40-7.
6. Ballert CS, Post MW, Brinkhof MW, Reinhardt JD. Psychometric properties of the Nottwil Environmental Factors Inventory Short Form. Arch Phys Med Rehabil. 2015;96(2):233-40.
7. National Health Survey: SF-36 Population Norms. Australian Bureau of Statistics; 1995.
8. Middleton J, Tate R, Geraghty T. Self-Efficacy and Spinal Cord Injury: Psychometric Properties of a New Scale. Rehabilitation Psychology. 2003;48(4):281-8.
9. Schwarzer R, Jerusalem M. Generalized Self-Efficacy scale. In: Weinman J, Wright S, Johnston M, editors. Measures in health psychology: A user's portfolio: Causal and control beliefs. Windsor, UK: NFER-NELSON; 1995. p. 35-7.
10. Sullivan M. User Manual for the Injustice Experience Questionnaire. Montreal, Quebec; 2008.

Supplementary Appendix 2: The ICF disability and health framework and self-reported QoL: supplementary and sensitivity analyses

Supplementary and sensitivity analyses	Key findings	TOTAL EFFECT<br>Estimate<br>(p value)	INDIRECT EFFECT<br>Estimate<br>(p value)	DIRECT EFFECT<br>Estimate<br>(p value)
**Sensitivity analysis 1:**<br>Alter the role of personal psychological factors<br>**Concept:** Treat self-efficacy and perceived injustice as underlying factors which influence perceptions about physical health, activity/participation problems and environmental factors<br>**Detail:** Self-efficacy and perceived injustice ***now lead to*** Physical health (SHC burden, pain), environmental factors (NEFI-S, social integration), activity/participation problem burden.<br>**Difference from the main path model (Figure 1):** The main path model represents self-efficacy and perceived injustice as mediating factors which are influenced by physical health, activity/participation problems and environmental factors.	*Self-efficacy sum score (per SD)*	0.36 (p<0.001)	0.27 (p<0.001)	0.09 (<0.001)
	Perceived injustice sum score (per SD)	-0.25 (p<0.001)	-0.20 (p<0.001)	-0.05 (0.03)
	Secondary health condition burden score (per SD)	-0.14 (p<0.001)	-0.15 (p<0.001)	0.007 (0.8)
	Maximum pain intensity (per SD)	-0.11 (p<0.001)	-0.05 (p<0.001)	-0.06 (0.007)
	NEFI-S score (per SD)	-0.06 (p=0.015)	-0.03 (p=0.02)	-0.04 (0.15)
	Social integration (per SD)	0.26 (p<0.001)	0.07 (p<0.001)	0.19 (<0.001)
	Activity/participation problem burden (per SD)	-0.20 (p<0.001)	-0.08 (p< 0.001)	-0.12 (<0.001)
**Sensitivity analysis 2:**<br>Alter the role of day-to-day assistance<br>**Concept:** Treat day-to-day assistance as a node (a separate box) which is affected by injury characteristics, functional independence level and physical health<br>**Detail:** Physical health (SHC burden, pain), functional independence level (m-SCIM-SR, used of a self-propelled manual wheelchair, use of an electric or assisted wheelchair), injury severity (complete lesion, tetraplegia) ***now lead to*** Any day-to-day assistance.<br>**Difference from the main path model (Figure 1):** In the main path model, day-to-day assistance was a covariable adjustment factor only	*Any day-to-day assistance*	-0.11 (p=0.07)	-0.09 (p=0.01)	-0.02 (0.7)
	Secondary health condition burden score (per SD)	-0.28 (p<0.001)	-0.29 (p<0.001)	0.01 (0.6)
	Maximum pain intensity (per SD)	-0.16 (p<0.001)	-0.10 (p<0.001)	-0.06 (0.006)
	m-SCIM-SR (per SD)	0.15 (p<0.001)	0.15 (p<0.001)	-0.004 (0.9)
	Use of electric or assisted wheelchair	0.03 (p=0.7)	0.06 (p=0.2)	-0.03 (0.6)
	Use of self-propelled manual wheelchair	0.14 (p=0.047)	0.09 (p=0.042)	0.05 (0.4)
	Complete lesion	-0.19 (p=0.007)	-0.02 (p=0.6)	-0.17 (<0.001)
	Tetraplegia	-0.13 (p=0.007)	-0.09 (p=0.01)	-0.04 (0.4)
**Sensitivity analysis 3:**<br>Add an effect of NEFI-S on social integration in 'Environmental'.<br>**Difference from the main path model (Figure 1):** In the main path model, relationships are specified between nodes (e.g. 'Environmental'), but not between factors within a node	*NEFI-S score (per SD)*	-0.22 (<0.001)	-0.18 (<0.001)	-0.04 (0.16)
	Social integration (per SD)	0.36 (<0.001)	0.17 (<0.001)	0.19 (<0.001)
**Sensitivity analysis 4:**<br>Add an effect of vitality on mental health in 'Energy and feelings'.<br>**Difference from the main path model (Figure 1):** In the main path model, relationships are specified between nodes (e.g. 'Energy and feelings') but not between factors within a node	*SF-36 Mental health domain score (per SD)*	0.18 (<0.001)	-0.001 (0.4)	0.18 (<0.001)
	SF-36 vitality domain score (per SD)	0.22 (<0.001)	0.08 (<0.001)	0.14 (<0.001)
**Supplementary analysis 1:**<br>Add 'Sleep problem severity' as a potential explanatory factor alongside SHC burden and pain within 'Physical health'. .	*Sleep problem severity (per SD)*	-0.17 (<0.001)	-0.13 (<0.001)	-0.05 (0.15)

**Difference from the main path model (Figure 1):** In the main path model, 'Physical health' comprises SHC and pain intensity		

Effects on QoL are expressed in terms of SDs of change.

Continuous variables included quality of life (QoL), self-efficacy sum score, perceived injustice sum score, secondary health condition (SHC) burden score, maximum pain intensity, Nottwil Environmental Factor Inventory Short Form (NEFI-S) score, social integration sum score, activity/participation problem burden score, SF-36 mental health domain score, SF-36 vitality domain score, the modified Spinal Cord Independence measure – Self Report (m-SCIM-SR), and sleep problem severity.

Dichotomous variables included receiving any day-to-day assistance, use of an electric or assisted wheelchair, use of a self-propelled manual wheelchair, having a complete lesion, and tetraplegia.

Standard deviation (SD), confidence interval (CI)
